# Supplementary material for: Bacterial polyphosphates interfere with the innate host defense to infection
Source: Nat Commun. 2020 Aug 12;11:4035. doi: 10.1038/s41467-020-17639-x (PMC7423913; doi:10.1038/s41467-020-17639-x)
Supplement: Supplementary file 1 — Supplementary Information [file 41467_2020_17639_MOESM1_ESM.pdf]

Supplementary Information

Bacterial Polyphosphates Interfere with the Innate Host Defense to Infection

Julian Roewe[1], Georgios Stavrides[1], Marcel Strueve[1], Arjun Sharma[1,2], Federico Marini[1,3], Amrit Mann[1], Stephanie A. Smith[4], Ziya Kaya[5], Birgit Strobl[6], Mathias Mueller[6], Christoph Reinhardt[1], James H. Morrissey[4], Markus Bosmann[1,2]

[1]Center for Thrombosis and Hemostasis, University Medical Center Mainz, 55131 Mainz, Germany.
[2]Pulmonary Center, Department of Medicine, Boston University School of Medicine, Boston, 02118, MA, USA.
[3]Institute of Medical Biostatistics, Epidemiology and Informatics, University Medical Center Mainz, 55131, Mainz, Germany.
[4]Department of Biological Chemistry, University of Michigan Medical School, Ann Arbor, 48109-1085, MI, USA.
[5]Department of Medicine III, University of Heidelberg, 69120 Heidelberg, Germany.
[6]Institute of Animal Breeding and Genetics, Department of Biomedical Science, University of Veterinary Medicine Vienna, 1210 Vienna, Austria.

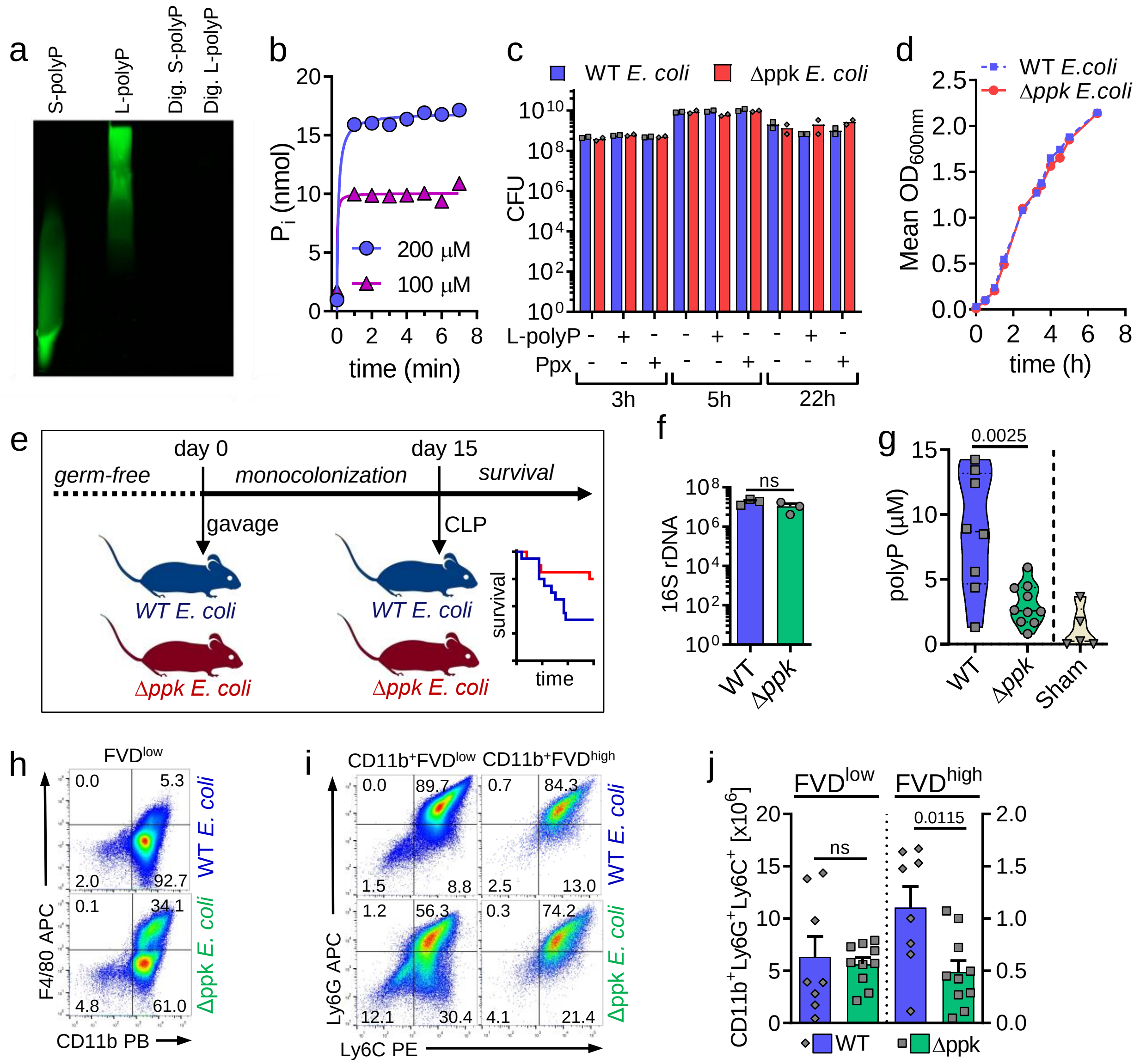

Supplementary Fig. 1 | Studies of polyphosphates, exopolyphosphatase and polyphosphatekinase-deficient (Δ*ppk*) *E. coli*. **a**, Gel electrophoresis followed by DAPI staining of intact short-chain polyphosphates (S-polyP, 5 μg/lane), long-chain polyphosphates (L-polyP, 5 μg/lane), or digested (dig.) S-polyP and L-polyP. Digestion was performed with 100 U/ml calf intestinal phosphatase (CIP) per sample for 24 h at 37°C. **b**, Enzymatic activity assay for recombinant Ppx purified from *S. cerevisiae*. L-polyP (100 μM or 200 μM) were added to a buffered solution of Ppx (27 μg/ml) and the appearance of inorganic monophosphates (P_i) as reaction product was detected by a colorimetric assay. **c**, *E. coli* strains were incubated as suspension cultures for the indicated time periods with L-polyP (200 μM), Ppx (3 μg/ml) or kept as untreated controls followed by assessment of viability (CFU) on agar plates. **d**, Bacterial growth of *E. coli* wild type (WT) and *E. coli* polyphosphatekinase-deficient (Δ*ppk*) was compared by optical densities (OD_{600nm}) of suspension cultures as a function of time. **e,** Schematic of the experimental design of *E. coli* monocolonization of germ-free mice followed by CLP sepsis. **f**, 16S rDNA copies by qPCR as surrogate for intestinal bacterial growth. Feces was collected 7 days after monocolonization of germ-free mice (n=3/group). The results suggested that *E. coli* Δ*ppk* retain bacterial fitness in a gut environment of monocolonization. **g**, Concentrations of polyphosphates in peritoneal lavage fluids 48 h after CLP of mice, which were monocolonized with WT *E. coli* (n=8) or Δ*ppk* *E. coli* (n=10). For assay validation, the peritoneal lavage concentrations of untreated healthy C57BL/6J mice (sham, n=5) are also shown. **h-i**, Flow cytometry of peritoneal $CD11b^+F4/80^+$ macrophages (**h**), live (FVD^{low}) and dead (FVD^{high}) $CD11b^+Ly6G^-Ly6C^+$ monocytes (**i**), and $CD11b^+Ly6G^+Ly6C^+$ neutrophils (**i**) from representative monocolonized mice 48 h after CLP; blots were pre-gated on $CD11b^+$ (CD11b-Pacific Blue). **j**, Total counts of live (FVD^{low}) and dead (FVD^{high}) neutrophils in peritoneal lavage of monocolonized mice (n=8 WT *E. coli*, n=10 Δ*ppk E. coli*) 48 h after CLP. Data are expressed as mean with s.e.m. as error bars and representative of two (**a**, **c**) or three (**b**, **d**) independent experiments., **f**, **g**, **j**: two-sided t-test, ns: not significant (p>0.05).

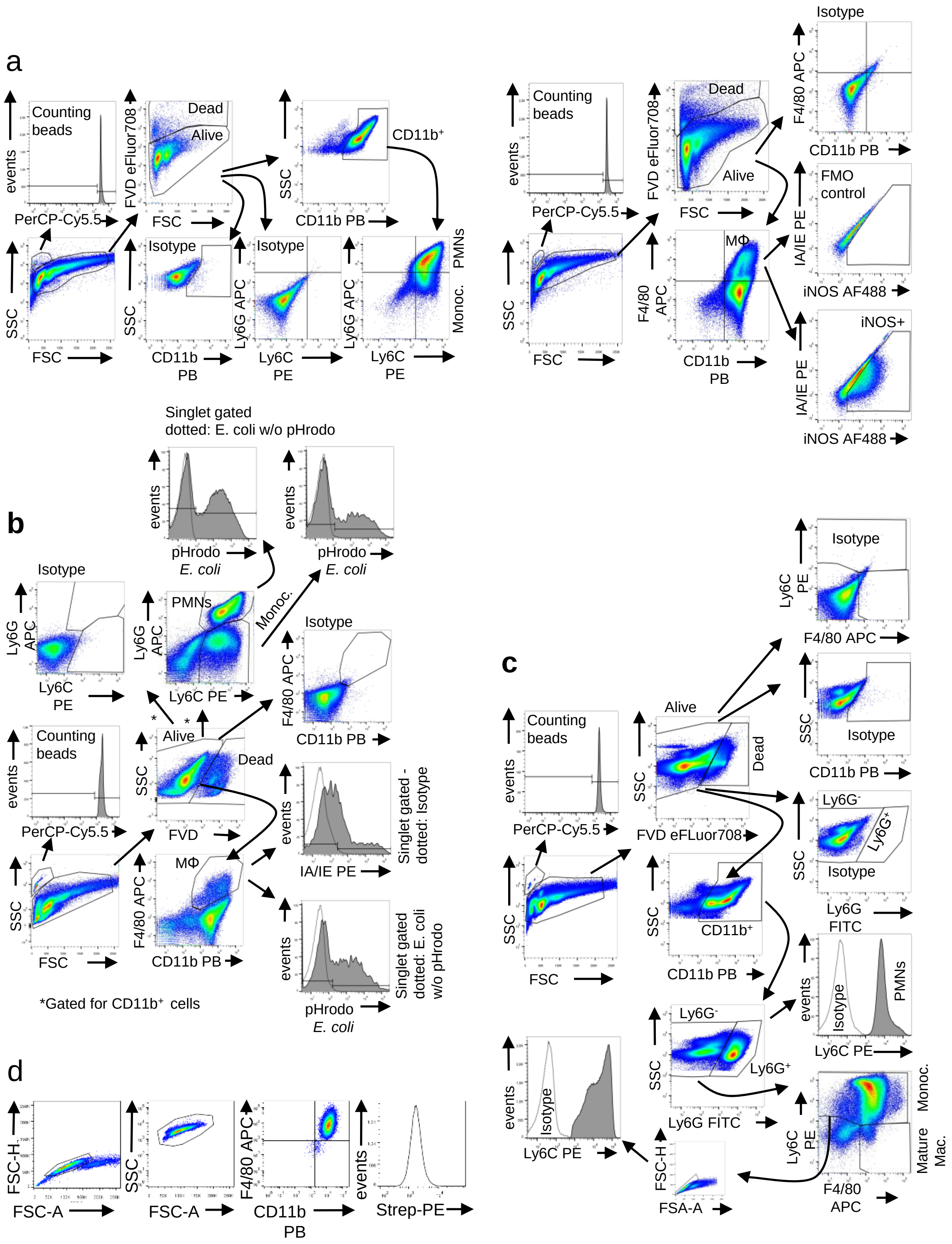

Supplementary Fig. 2 | Representative gating strategies for flow cytometry. a, Myeloid cells from peritoneal lavages of CLP sepsis experiments were stained with a fixable viability dye (FVD eFluor780), antibodies for surface markers and iNOS used together with isotype controls plus addition of counting beads for calculation of absolute cell numbers. **b**, Phagocytosis assay of myeloid cells after intra-peritoneal injection of green-fluorescent pHrodo-*E. coli* bioparticles. **c**, Myeloid cells from peritoneal lavages following thioglycolate-induced migration. **d**, Macrophages (BMDM) stained with surface markers CD11b, F4/80 and biotinylated L-polyphosphates / streptavidin-PE. FSC: forward scatter, SSC: side scatter.

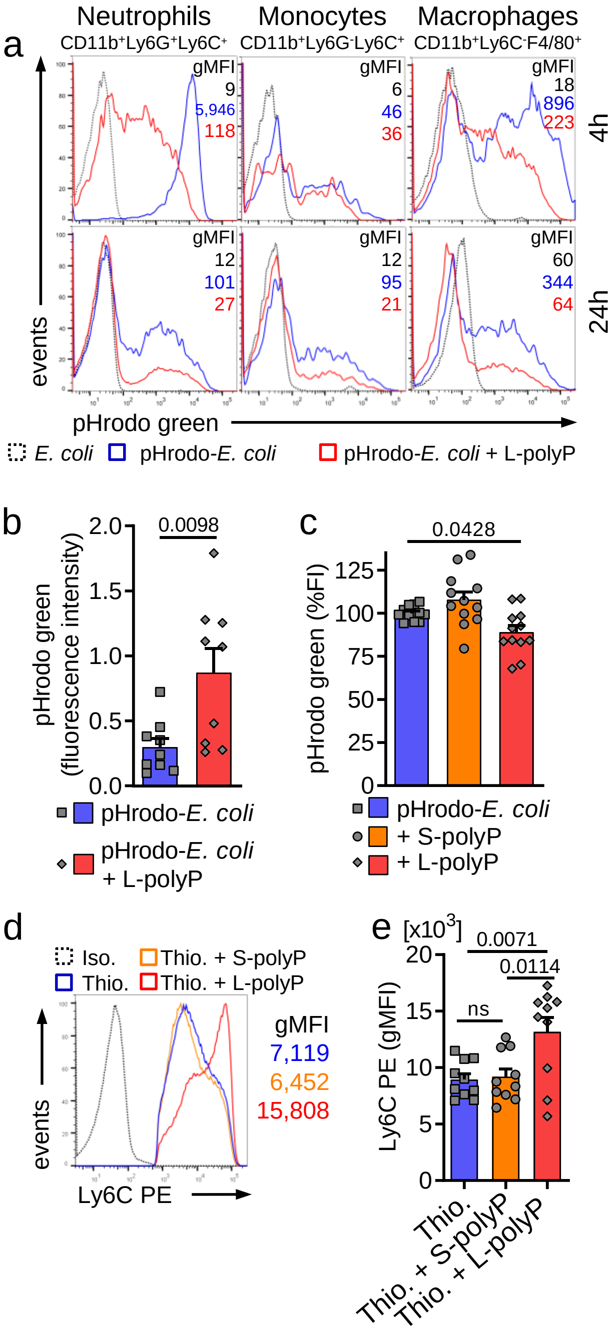

Supplementary Fig. 3 | Polyphosphate-dependent modulation of myeloid cell responses. a, Phagocytic activities of peritoneal neutrophils, monocytes and macrophages 4 h or 24 h following intra-peritoneal injection of fluorescent reporter pHrodo-*E. coli* bioparticles ± long-chain polyphosphates (L-polyP: 10 µg/g BW) into C57BL/6J mice shown as flow cytometry histograms. Unlabelled *E. coli* particles served as negative controls (staining 1: FVD eFluor780, CD11b-Pacific Blue, F4/80-APC, I-A/I-E-PE, pHrodo green; staining 2: FVD eFluor 780, CD11b-Pacific Blue, Ly6G-APC, Ly6C-PE, pHrodo green). **b**, Fluorescence of non-ingested pHrodo-*E. coli* in eukaryotic cell-free peritoneal lavages (as in frame a), when treated with acidified buffer for reporter activation (n=9 mice/group). **c**, Phagocytosis of pHrodo-*E. coli* by cultured macrophages (BMDM, C57BL/6J) depicted as normalized fluorescence intensities, 6 h, n=12 samples/group. **d**, Ly6C on peritoneal $CD11b^{+}Ly6G^{-}$ monocytes 24 h after intra-peritoneal (i.p.) injection of thioglycolate (Thio.) ± S-/L-polyP (10 µg/g BW) in C57BL/6J mice shown as representative flow cytometry histograms pre-gated on $CD11b^{+}$, Iso.: Isotype control antibody, gMFI: geometric mean fluorescence intensity (FVD eFluor 780, CD11b-Pacific Blue, F4/80-APC, Ly6G-FITC, Ly6C-PE). **e**, Ly6C fluorescence on monocytes pooled from n=10 mice/group from experiments as in frame d. Data are expressed as mean ± s.e.m., **b**: two-sided t-test, **c**, **e**: one-way ANOVA, ns: not significant (p>0.05).

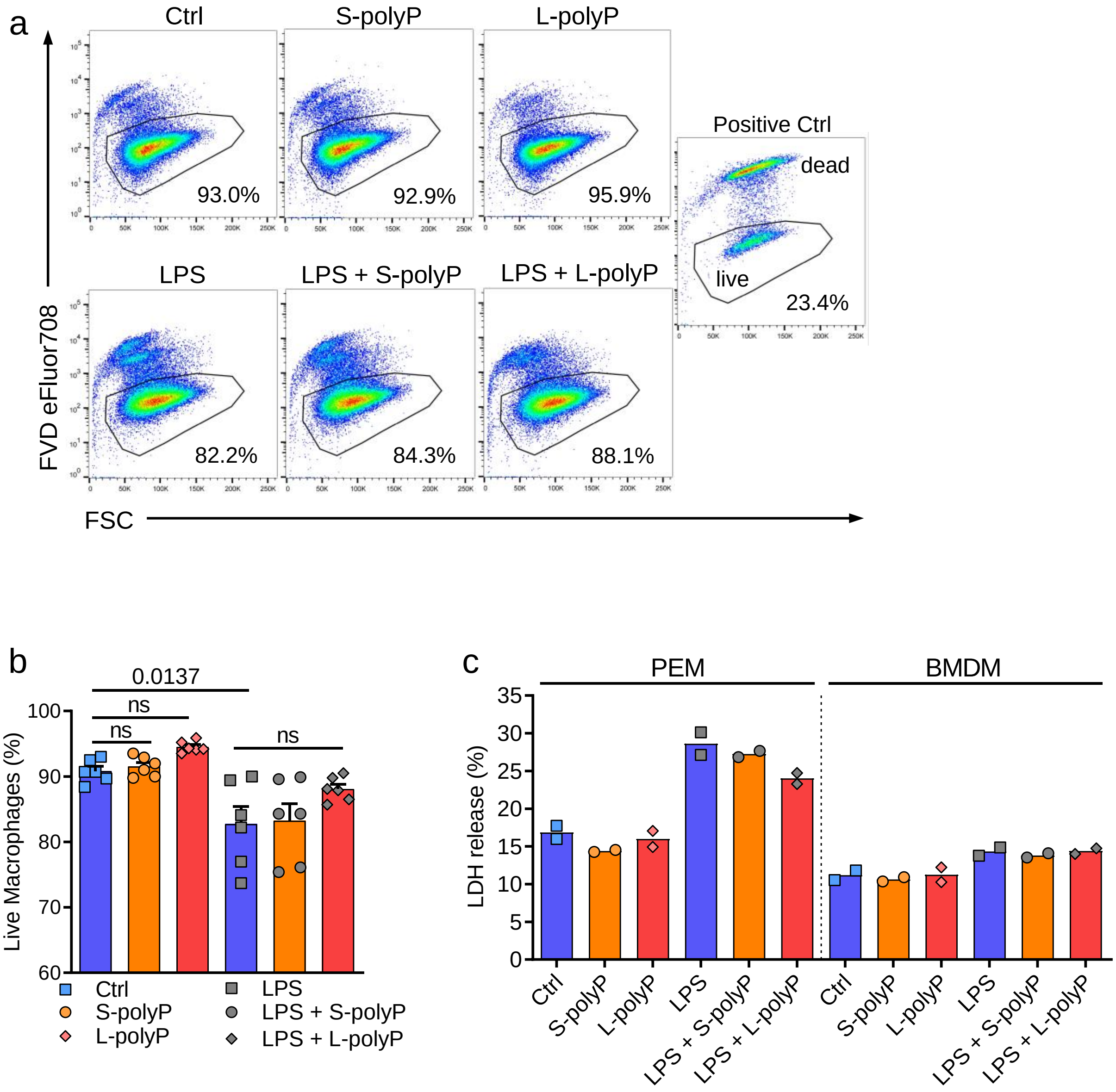

Supplementary Fig. 4 | Polyphosphates and macrophage viability. **a**, Evaluation of polyphosphate associated toxicity using a fixable viability dye (FVD eFluor780) in flow cytometry. BMDM (C57BL/6J) were stimulated 24 h with LPS (100 ng/ml) ± short-chain or long-chain polyphosphates (S-/L-polyP: 50 µM), S-/L-polyP alone, or left as untreated controls (Ctrl). Positive control: heat-killed BMDM (1 min at 65°C) were mixed with untreated cells, FSC: forward scatter. **b**, Pooled viability data (n=6 samples/group) examined over three independent experiments as in frame a. **c**, Lactatdehydrogenase (LDH) release to culture supernatants as surrogate for viability of peritoneal macrophages (PEM) or BMDM treated as in frame a. LDH activity was normalized to lysed cell samples (=100%). Data are representative of two independent experiments. **b**, **c**: Data are expressed as mean ± s.e.m., one-way ANOVA, ns: not significant (p>0.05).

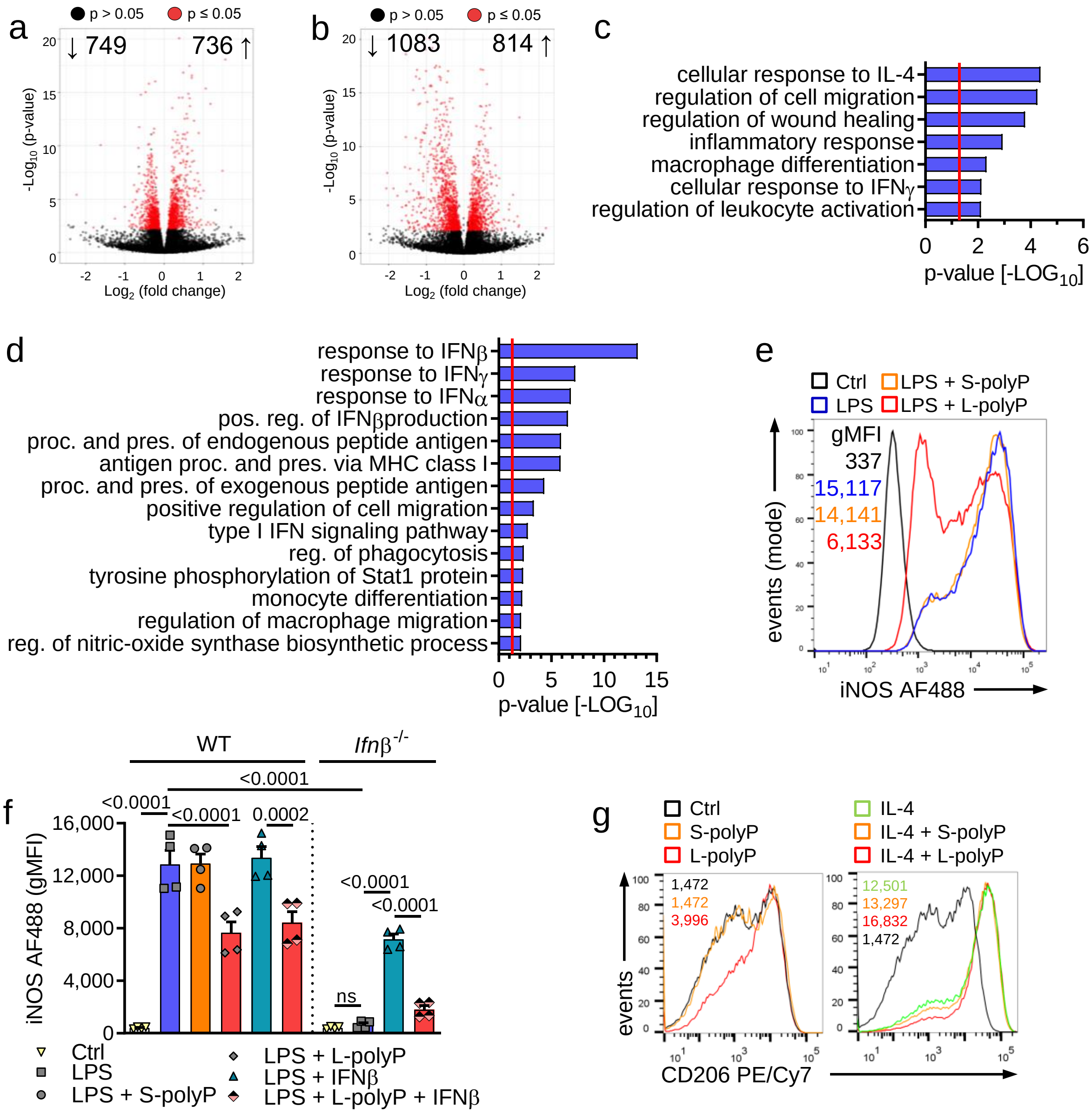

Supplementary Fig. 5 | Polyphosphate-dependent regulation of macrophage transcriptomes and validation studies of selected proteins. a-b, Volcano plots of RNA-seq data from macrophages of long-chain polyphosphates (L-polyP) vs. untreated controls (Ctrl) in frame (a) or LPS vs. LPS+L-polyP in frame (b). DEGs (adjusted p≤0.05) are shown as red dots together with total counts of up-regulated DEGs (↑) and down-regulated DEGs (↓), BMDM, C57BL/6J, n=5/group. **c-d**, Selection of overrepresented pathways in DEG datasets using GO enrichment analysis comparing L-polyP vs. Ctrl (c) and LPS+L-polyP vs. LPS (d). p=0.05 is denoted as a red line. **e**, Representative histograms (for data in Fig. 3d) of intracellular iNOS protein in CD11b$^+$F4/80$^+$ macrophages (FVD eFluor 780, CD11b-Pacific Blue, F4/80-APC iNOS-Alexa Fluor 488) at 24 h after LPS ± S-/L-polyP stimulation as compared to unstimulated control cells (Ctrl). **f**, Intracellular iNOS as geometric mean fluorescence intensities (gMFI) of CD11b$^+$F4/80$^+$ macrophages from wild type (WT) and IFNβ-deficient (*Ifnβ*$^{-/-}$) mice 24 h after LPS ± S-/L polyP (n=4 samples/group). The rmIFNβ (500 U/ml) was added 3 h after LPS, where indicated. Data are pooled from representative two of three independent experiments (FVD eFluor 780, CD11b-Pacific Blue, F4/80-APC, iNOS-Alexa Fluor 488). **g**, Representative histograms (for data in Fig. 3h-i) of CD206 presence in CD11b$^+$F4/80$^+$ macrophages preincubated with or without rmIL-4 for 24 h followed by S-/L-polyP for additional 24 h and controls (FVD eFluor 780, CD11b-Pacific Blue, F4/80-APC, CD206-PE/Cy7). S-/L-polyP: 50 µM, LPS: 100 ng/ml. **f**: Data are expressed as mean ± s.e.m., one-way ANOVA, ns: not significant (p>0.05).

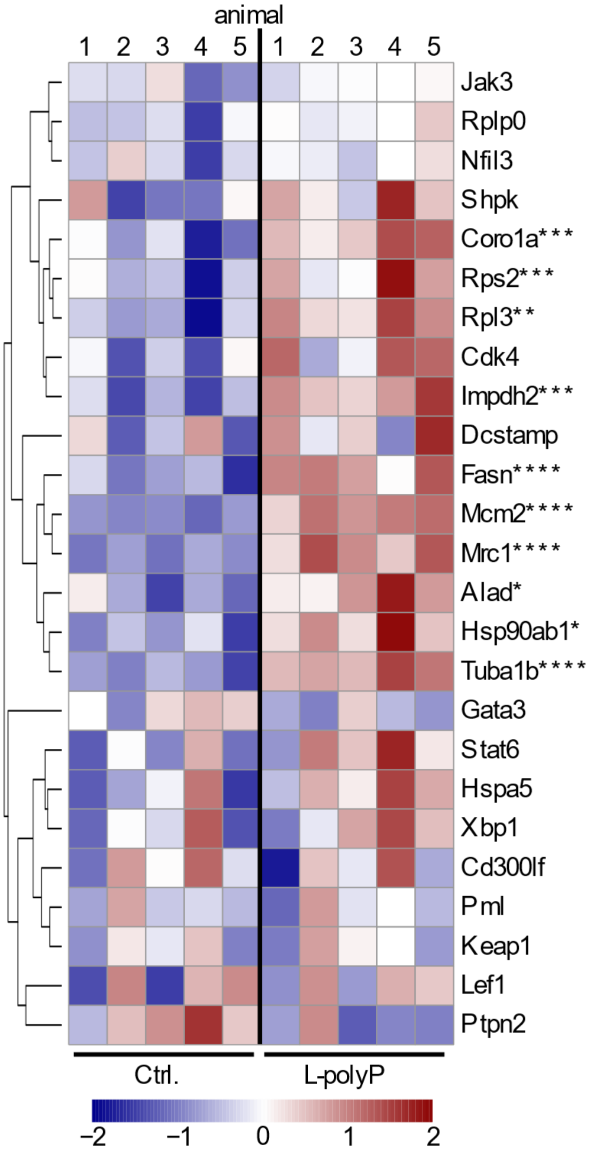

Supplementary Fig. 6 | Polyphosphates modulate the expression of IL-4 response genes in macrophages. Heatmap of normalized expression (Z-score from +2/red to -2/blue) of IL-4 response associated genes (GO: 0071353) from RNA-seq data of macrophages (BMDM, n=5 samples/group) comparing long-chain polyphosphates (L-polyP) to unstimulated controls (Ctrl). Adjusted p-values (FDR correction) of DEGs (DESeq 2 analysis) are marked by asterisks, *p<0.05, **p<0.01, ***p<0.001, ****p<0.0001.

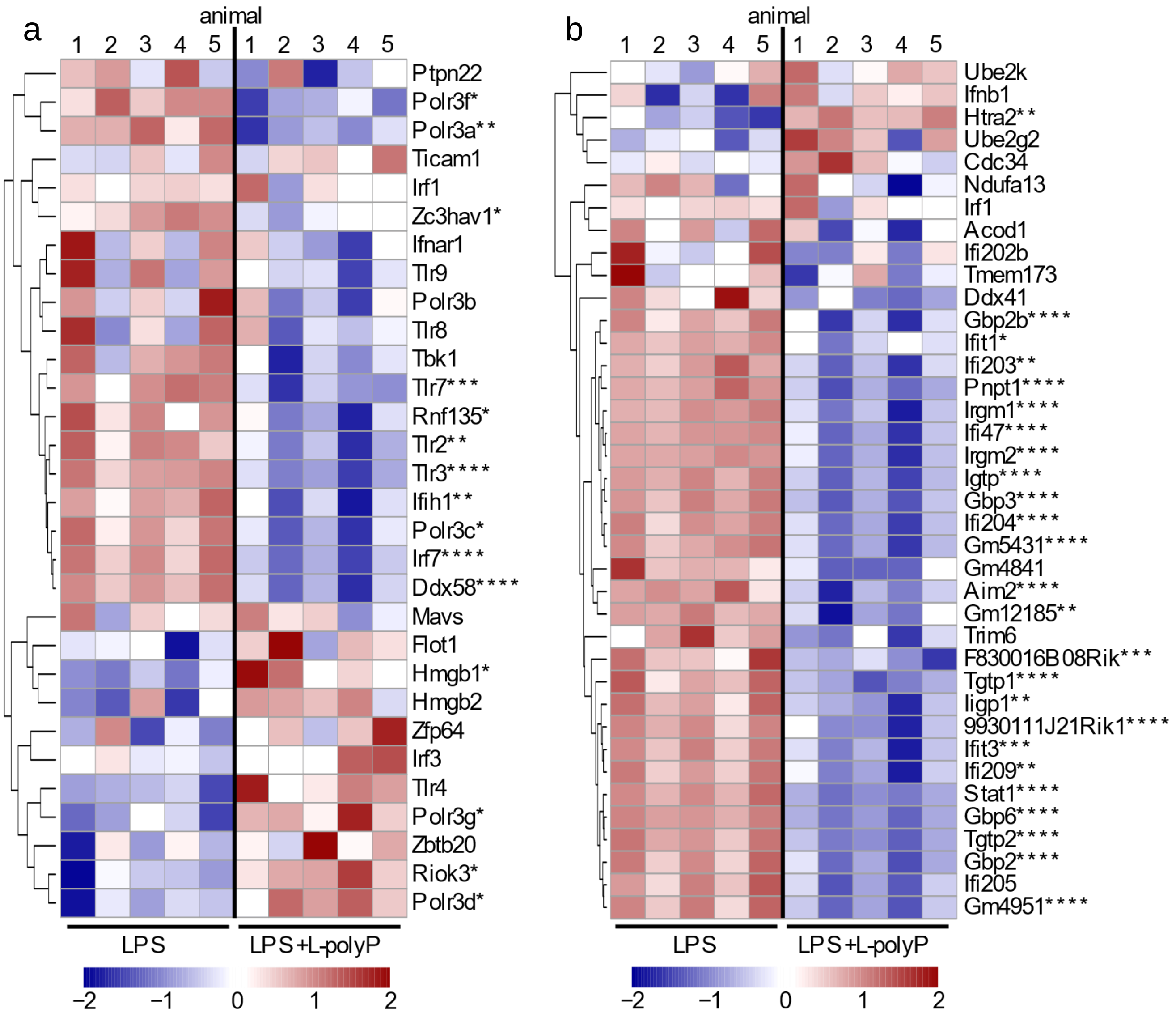

Supplementary Fig. 7 | Polyphosphates restrain IFNβ pathways of LPS/TLR4-activated macrophages. **a**, Genes associated with the pathway of positive regulation of IFNβ production (GO: 0032728). **b**, Genes associated with response to IFNβ (GO: 0035456). Data are from RNA-seq of macrophages (BMDM, n=5 samples/group) comparing LPS + long-chain polyphosphates (L-polyP) vs. LPS alone. Heatmaps of normalized expression values (Z-score from +2/red to -2/blue) and adjusted p-values (FDR correction) of DEGs (DESeq 2 analysis) are marked by asterisks, *p<0.05, **p<0.01, ***p<0.001, ****p<0.0001.

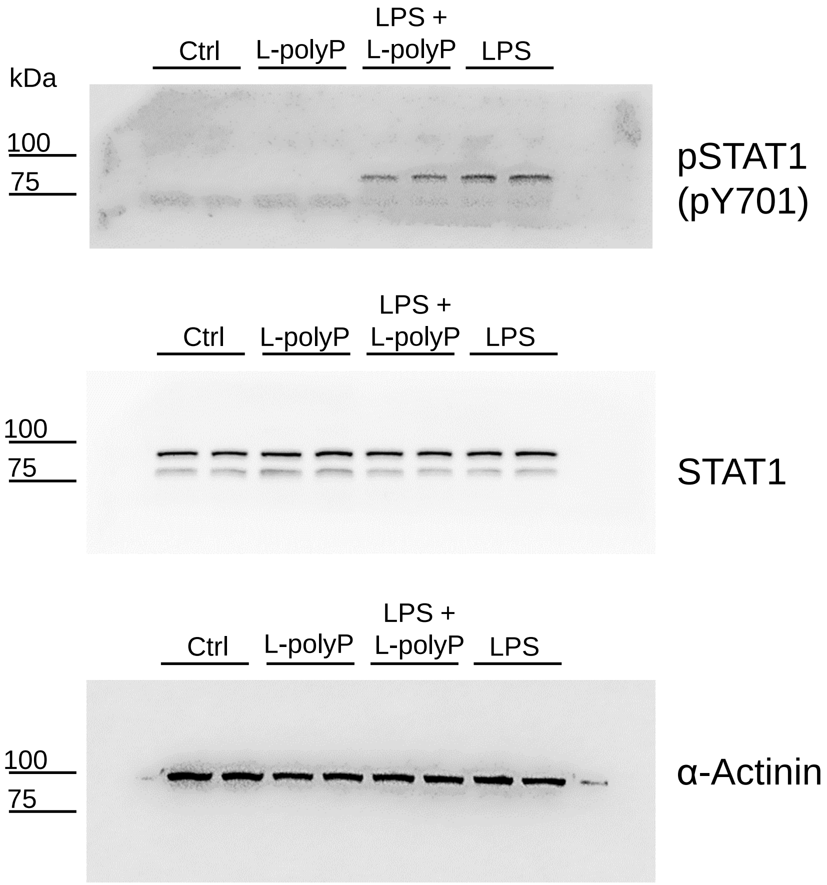

Supplementary Fig. 8 | Full images of western blots. Macrophages (C57BL/6J, BMDM) were incubated as technical duplicates with LPS (100 ng/ml) and L-polyP (50 µM) for 3 h or kept as untreated controls (Ctrl). Cell lysates were analysed by western blotting with antibodies against phosphorylated STAT1^{Y701} (top), total STAT1 (center), and housekeeping gene α-Actinin (bottom). The images represent the full uncroped blots of data as shown in Fig. 4i.

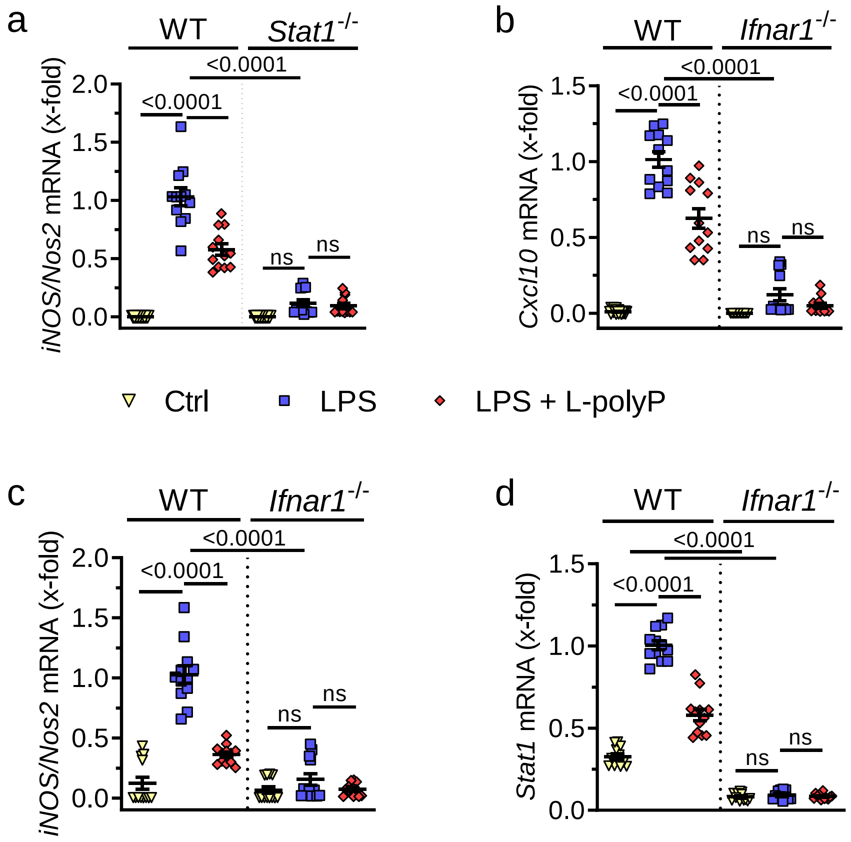

Supplementary Fig. 9 | Roles of IFNAR and STAT1 for polyphosphate-dependent gene regulation.
a, RT-qPCR analysis of iNOS mRNA from macrophages (BMDM) derived from *Stat1*$^{-/-}$ mice as compared to wild type (WT) mice. Macrophages were stimulated for 12 h with LPS (100 ng/ml) ± long-chain polyphosphates (L-polyP: 50 µM) or kept as untreated controls (Ctrl). **b-d**, RT-qPCR analysis of CXCL10 (**b**), iNOS (**c**), and STAT1 (**d**) of macrophages (n=12 samples/group) from *Ifnar1*$^{-/-}$ or corresponding wild type mice as in frame a. Data in each frame are pooled from n=12 samples/group examined over three independent experiments and expressed as mean ± s.e.m., one-way ANOVA, ns: not significant (p>0.05).

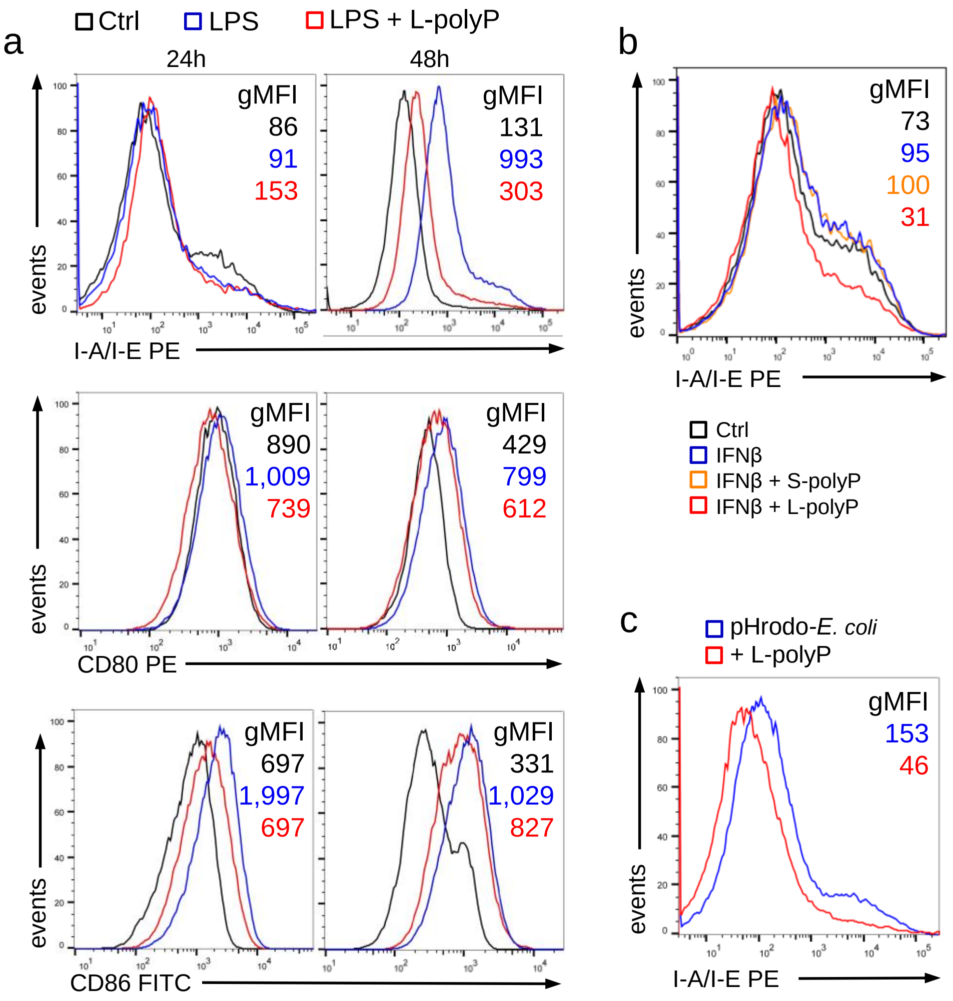

Supplementary Fig. 10 | Interference of polyphosphates with MHC class II antigen presentation in macrophages. a, Flow cytometry analysis showing histograms of MHCII (I-A/I-E; top panel; FVD eFluor780, CD11b-Pacific Blue, F4/80-APC, I-A/I-E-PE), CD80 (center panel; FVD eFluor780, CD11b-Pacific Blue, F4/80-APC, CD80-PE), and CD86 (lower panel; FVD eFluor780, CD11b-Pacific Blue, F4/80-APC, CD86-FITC) surface expression on CD11b$^+$F4/80$^+$ BMDM 24 h (left) and 48 h (right) after LPS ± long-chain polyphosphate (L-polyP) stimulation and resting cells (Ctrl). **b**, Representative histograms of MHCII (I-A/I-E) surface expression on CD11b$^+$F4/80$^+$ BMDM 24 h after rmIFNβ with or without 3 h pre-incubation of S-/L-polyP. Data of frames a-b are representative of three independent experiments (FVD eFluor780, CD11b-Pacific Blue, F4/80-APC, I-A/I-E-PE). **c**, Representative (of n=9/group) histograms of MHCII (I-A/I-E) surface expression on CD11b$^+$F4/80$^+$ peritoneal macrophages isolated 24 h after intra-peritoneal injections of pHrodo-*E. coli* particles ± L-polyP (FVD eFluor780, CD11b-Pacific Blue, F4/80-APC, I-A/I-E-PE).

Supplementary Table 1 | Primer sequences (*M. musculus*)

Target (orientation)	5' – 3'	Reference
***16S* rDNA (forward)**	GTGSTGCAYGGYYGTCGTCA	[1]
**16S rDNA (reverse)**	ACGTCRTCCMCNCCTTCCTC	[1]
***Arg1* (forward)**	CAGAAGAATGGAAGAGTCAG	[2]
***Arg1* (reverse)**	CAGATATGCAGGGAGTCACC	[2]
***Ciita* (forward)**	GAGATCCCAGATCCATGGTG	[3]
***Ciita* (reverse)**	CTCTCTAAATCATGCGCTGC	[3]
***Cxcl10* (forward)**	AGGAGCCCTTTTAGACCTTTTTTG	[3]
***Cxcl10* (reverse)**	CACCATGAACCCAAGTGCTGCCGT	[3]
***Fizz1* (forward)**	TCCCAGTGAATACTGATGAGA	[2]
***Fizz1* (reverse)**	CCACTCTGGATCTCCCAAGA	[2]
***Gapdh* (forward)**	TACCCCCAATGTGTCCGTCGTG	[3]
***Gapdh* (reverse)**	CCTTCAGTGGGCCCTCAGATGC	[3]
***Ifi44* (forward)**	GTTCGGATGGTTTGATGTGA	[3]
***Ifi44* (reverse)**	GCACACAGACGATGTATGGC	[3]
***Ifit1* (forward)**	GACCTGGTCACCATCAGCAT	[3]
***Ifit1* (reverse)**	CAAGGCAGGTTTCTGAGGAG	[3]
***iNos/Nos2* (forward)**	TTCTGTGCTGTCCCAGTGAG	[3]
***iNos/Nos2* (reverse)**	TGAAGAAAACCCCTTGTGCT	[3]
***Irf7* (forward)**	AGCATTGCTGAGGCTCACTT	[3]
***Irf7* (reverse)**	TGATCCGCATAAGGTGTACG	[3]
***Msc4ac* (forward)**	GGGCAGCAGAAACAGTACAGA	[3]
***Msc4ac* (reverse)**	CGATGGGTGAGAACACACAA	[3]
***Nlrc5* (forward)**	CTTCCCGCCTCTCCTTCCACAAT	[4]
***Nlrc5* (reverse)**	CTCCACCTGCCCACATCCTACCA	[4]
***Rfx5* (forward)**	ATAATGACCGTTCTCGAGGG	[3]
***Rfx5* (reverse)**	CAGCAGCATCTCATCTCTGC	[3]
***Socs1* (forward)**	ACAAGCTGCTACAACCAGGG	[3]
***Socs1* (reverse)**	ACTTCTGGCTGGAGACCTCA	[3]
***Stat1* (forward)**	CTGAATATTTCCCTCCTGGG	[3]
***Stat1* (reverse)**	TCCCGTACAGATGTCCATGAT	[3]
***Ym1* (forward)**	GGGCATACCTTTATCCTGAG	[2]
***Ym1* (reverse)**	CCACTGAAGTCATCCATGTC	[2]

Supplementary References

1 Fuller, Z. *et al.* Influence of cabbage processing methods and prebiotic manipulation of colonic microflora on glucosinolate breakdown in man. *Br J Nutr* **98**, 364-372, doi:10.1017/S0007114507709091 (2007).

2 Wang, J. *et al.* Bacterial colonization dampens influenza-mediated acute lung injury via induction of M2 alveolar macrophages. *Nature communications* **4**, 2106, doi:10.1038/ncomms3106 (2013).

3 Cui, W., Taub, D. D. & Gardner, K. qPrimerDepot: a primer database for quantitative real time PCR. *Nucleic Acids Res* **35**, D805-809, doi:10.1093/nar/gkl767 (2007).

4 Biswas, A., Meissner, T. B., Kawai, T. & Kobayashi, K. S. Cutting edge: impaired MHC class I expression in mice deficient for Nlrc5/class I transactivator. *Journal of immunology* **189**, 516-520, doi:10.4049/jimmunol.1200064 (2012).
