# Supplementary material for: Bacterial polyphosphates interfere with the innate host defense to infection
Source: Nat Commun. 2020 Aug 12;11:4035. doi: 10.1038/s41467-020-17639-x (PMC7423913; doi:10.1038/s41467-020-17639-x)
Supplement: Supplementary file 3 — Reporting Summary [file 41467_2020_17639_MOESM3_ESM.pdf]

nature research

Corresponding author(s): Markus Bosmann

Last updated by author(s): May 29, 2020

Reporting Summary

Nature Research wishes to improve the reproducibility of the work that we publish. This form provides structure for consistency and transparency in reporting. For further information on Nature Research policies, see Authors & Referees and the Editorial Policy Checklist .

Statistics

For all statistical analyses, confirm that the following items are present in the figure legend, table legend, main text, or Methods section.

n/a	Confirmed	
☐	☒	The exact sample size (*n*) for each experimental group/condition, given as a discrete number and unit of measurement
☐	☒	A statement on whether measurements were taken from distinct samples or whether the same sample was measured repeatedly
☐	☒	The statistical test(s) used AND whether they are one- or two-sided<br>*Only common tests should be described solely by name; describe more complex techniques in the Methods section.*
☒	☐	A description of all covariates tested
☐	☒	A description of any assumptions or corrections, such as tests of normality and adjustment for multiple comparisons
☐	☒	A full description of the statistical parameters including central tendency (e.g. means) or other basic estimates (e.g. regression coefficient) AND variation (e.g. standard deviation) or associated estimates of uncertainty (e.g. confidence intervals)
☐	☒	For null hypothesis testing, the test statistic (e.g. *F*, *t*, *r*) with confidence intervals, effect sizes, degrees of freedom and *P* value noted<br>*Give P values as exact values whenever suitable.*
☒	☐	For Bayesian analysis, information on the choice of priors and Markov chain Monte Carlo settings
☒	☐	For hierarchical and complex designs, identification of the appropriate level for tests and full reporting of outcomes
☒	☐	Estimates of effect sizes (e.g. Cohen's *d*, Pearson's *r*), indicating how they were calculated

Our web collection on statistics for biologists contains articles on many of the points above.

Software and code

Policy information about availability of computer code

Data collection	BD FACSDiva Software v6.13<br>Fusion FX v16.11<br>Revelation G3.2<br>Ascent Software v2.6<br>CFX Manager v3.1
Data analysis	FlowJo v10<br>Graphpad Prism v8.0<br>STAR aligner v2.4.0b<br>pcaExplorer package v2.2.1<br>FastQC v0.11.2<br>DESeq2 v1.16.0<br>topGO v2.28.0<br>goseq v1.28.9

For manuscripts utilizing custom algorithms or software that are central to the research but not yet described in published literature, software must be made available to editors/reviewers. We strongly encourage code deposition in a community repository (e.g. GitHub). See the Nature Research guidelines for submitting code & software for further information.

Data

Policy information about availability of data

All manuscripts must include a data availability statement. This statement should provide the following information, where applicable:

- Accession codes, unique identifiers, or web links for publicly available datasets
- A list of figures that have associated raw data
- A description of any restrictions on data availability

The RNA-seq data presented in this manuscript have been deposited in the Gene Expression Omnibus (GEO) under accession number GSE131561. All other data are available from the corresponding author upon reasonable request.

Field-specific reporting

Please select the one below that is the best fit for your research. If you are not sure, read the appropriate sections before making your selection.

- [x] Life sciences
- [ ] Behavioural & social sciences
- [ ] Ecological, evolutionary & environmental sciences

For a reference copy of the document with all sections, see nature.com/documents/nr-reporting-summary-flat.pdf

Life sciences study design

All studies must disclose on these points even when the disclosure is negative.

Sample size	The samples sizes were determined by power calculations with estimating the effect sizes based on previous experiences and publications (PMID28835457, PMC3904521).
Data exclusions	From the RNA-seq study one sample of macrophages was excluded due to different sex of the donor and a strong gender effect for the clustering of the results (remaining sample size was n=5/group). For other studies, exclusion of outliers was only allowed if it was obviously caused by incorrectly entered or measured data. Exclusion criteria were pre-established.
Replication	Individual values in figures represent the numbers of mice for in vivo experiments and in vitro experiments were performed as three independent biological replicates with technical duplicates unless stated otherwise in figure legends.
Randomization	1. Age- and gender-matched mice were randomly allocated to the different experimental groups for in vivo experiments.<br>2. Randomization was not relevant for cell culture experiments because identical cells were used for all treatment conditions of an experimental series.<br>3. Randomization was not feasible for allocation of groups based on their genotype. In these cases, the groups were on matched genetic backgrounds to minimize covariates.
Blinding	Surgeons were blinded to group allocation for CLP sepsis and post-interventional care of mice. Investigators were not blinded for cell culture experiments or data analysis as this was not relevant to the study because of no risk for an observer bias.

Reporting for specific materials, systems and methods

We require information from authors about some types of materials, experimental systems and methods used in many studies. Here, indicate whether each material, system or method listed is relevant to your study. If you are not sure if a list item applies to your research, read the appropriate section before selecting a response.

Materials & experimental systems

n/a	Involved in the study	
[ ]	[x]	Antibodies
[ ]	[x]	Eukaryotic cell lines
[x]	[ ]	Palaeontology
[ ]	[x]	Animals and other organisms
[x]	[ ]	Human research participants
[x]	[ ]	Clinical data

Methods

n/a	Involved in the study	
[x]	[ ]	ChIP-seq
[ ]	[x]	Flow cytometry
[x]	[ ]	MRI-based neuroimaging

Antibodies

Antibodies used	Flow Cytometry<br>• CD16/CD32 Fc-block (clone 93) Biolegend #101329<br>• CD11b (clone M1/70), Pacific Blue, Biolegend #101224<br>• Ly6G (clone 1A8), Allophycocyanin and Fluorescein Isothiocyanate, Biolegend #127614 & #127606

• Ly6C (clone HK1.4), Phycoerythrin, Biolegend #128008
• F4/80 (clone BM8), Allophycocyanin Biolegend #123116
• I-A/I-E (clone M5/114.15.2), Phycoerythrin, Biolegend #107608
• CD80 (clone 16-10A1), Phycoerythrin, Biolegend #104708
• CD86 (clone GL-1), Fluorescein Isothiocyanate, Biolegend #105006
• CD206 (clone C068C2), Phycoerythrin-Cyanine7, Biolegend #141720
• iNOS/NOS2 (clone CXNFT), Alexa Fluor 488, Thermo Fisher Scientific #53-5920-82
• phospho-STAT1(Y701) (clone 4a), Phycoerythrin, BD Bioscience #612564
WB
• α-actinin (polyclonal), Cell Signaling Technology #3134
• STAT1 (polyclonal), Cell Signaling Technology #9172S
• phospho-STAT1(Y701) (clone 58D6), Cell Signaling Technology #9167S
• horseradish peroxidase conjugated anti-rabbit IgG, Vector Laboratories #PI-1000-1

Validation

All antibodies used in flow cytometry experiments were tested against the matched isotype controls
Flow Cytometry
• CD16/CD32 Fc-block (clone 93) Biolegend #101329:
AB_2783037
Petursdottir D, et al. 2017. Front Immunol. 10.3389/fimmu.2017.01699; Tong Y, et al. 2018. EBioMedicine. 39:132; Guérin MV, et al. 2019. Nat Commun. 10:4131; Le TM et al. 2018. Journal of neurochemistry. 145(2):139-153; Schmidleithner L et al. 2019. Immunity. 50(5):1232-1248; Lercher A, et al. 2019. Immunity. 51:1074
• CD11b (clone M1/70), Pacific Blue, Biolegend #101224
AB_755985
Taherzadeh Z, et al. 2010. Am J Physiol Heart Circ Physiol. 298:H1273; Smith K, et al. 2012. Infect Immun . 80:3481; Janelsins B, et al. 2013. Blood. 121:2923; Raines A, et al. 2013. Development. 140:2942; Sharma A, et al. 2014. J Infect Dis.; Roberts L, et al. 2014. Infect Immun . 82:2504; Endo J, et al. 2014. J Exp Med. 211:1673; Ni P, et al. 2014. J Immunol . 193:1778; Huang A, et al. 2014. Exp Cell Res. 15:326; Stienstra R, et al. 2014. Diabetes. 63:4143; Nacer A, et al. 2014. PLoS Pathog. 10:1004528; Krishnamoorthy N, et al. 2015. J Immunol. 194:863a
• Ly6G (clone 1A8), Allophycocyanin and Fluorescein Isothiocyanate, Biolegend #127614 & #127606
AB_2227348
Gottfried-Blackmore A, et al. 2009. Proc Natl Acad Sci U S A. 106:20918; Oertli M, et al. 2011. J Immunol. 187:3578; Kamei A, et al. 2012. J Infect Dis. 207:39; Devi S, et al. 2013. J Exp Med. 210:2321; Sharma A, et al. 2014. J Infect Dis.; Patnode M, et al. 2014. J Exp Med. 211:1281; Zhou Q, et al. 2014. J Immunol. 193:496; Christian D, et al. 2014. Infect Immun . 82:4056; Lee M, et al. 2014. PLoS One. 9:112666; Cohen M, et al. 2014. EMBO J. 33:2906; Price P, et al. 2015. J Immunol. 194:1164; Steichen A, et al. 2015. PLoS One. 10:123573
AB_1236494
Simpson K, et al. 2012. J Immunol. 189:5533; Fujikura D, et al. 2013. PLoS One. 8:e55321; Wu M, et al. 2013. Proc Natl Acad Sci U S A. 110:9439; Jacome-Galarza C, et al. 2011. J Bone Miner Res. 26:1207; Gorina R, et al. 2014. J Immunol. 192:324; Abad C, et al. 2014. J Leukoc Biol. 95:357; Weinheimer-Haus E, et al. 2014. PLoS One. 9:91355; Xu Y, et al. 2014. Proc Natl Acad Sci U S A. 111:6371; Mandraju R, et al. 2014. J Immunol. 192:4303; Minogue A, et al. 2014. Neurobiol Aging. 35:1442; Chen J, et al. 2014. Cell Res. 24:1050; Ericson J, et al. 2014. PLoS One. 9:108553
• Ly6C (clone HK1.4), Phycoerythrin, Biolegend #128008
AB_1186132
Zuber J, et al. 2009. Genes Dev. 1.567361111; Younos I, et al. 2011. Int Immunopharmacol. 11:816; Petersen B, et al. 2014. J Leukoc Biol. 95:809; Radovanovic I, et al. 2014. J Immunol. 193:1290; Chen J, et al. 2014. Cell Res. 24:1050; Koronyo Y, et al. 2015. Brain. 138: 2399-2422; Chen S, et al. 2015. Cancer Res . 7: 519-531; Zuchtriegel G, et al. 2016. PLoS Biol. 14: 1002459; Kar S, Colino J, Snapper C 2016. J Immunol. 196: 4204 - 4213; Denk F, et al. 2016. Cell Rep. 15: 1771-1781; Gousopoulos E, et al. 2016. Am J Pathol. 186:2193-2203; Francis M, et al. 2017. Toxicol Sci. 155(1):182-195
• F4/80 (clone BM8), Allophycocyanin Biolegend #123116
AB_893481
Prantner D, et al. 2009. Infect Immun. 77:5334; Sun L, et al. 2009. FASEB J. 3.602777778; Oatley J, et al. 2009. Development. 136:1191; Karpurapu M, et al. 2011. Blood. 118:5255; Prantner D, et al. 2011. Infect Immun. 79:3922; Kim M, et al. 2012. J Immunol. 188:4158; Malik M, et al. 2012. Proc Natl Acad Sci U S A. 109:6130; Klezovich-Bénard M, et al. 2012. PLoS One. 8:e1002481; Stubelius A, et al. 2012. Immunobiology. 217:751; Neimert-Andersson T, et al. 2011. Vaccine. 29:8965; Horsle B 2013. Development. 140:1517; Patankar Y, et al. 2013. Infect Immun . 81:2043
• I-A/I-E (clone M5/114.15.2), Phycoerythrin, Biolegend #107608
AB_313323
Yasmin N, et al. 2013. J Exp Med. 210:2597; Xia S, et al. 2014. J Leukoc Biol. 95:733; Markey K, et al. 2014. J Immunol. 192:5426; Sakai F, et al. 2014. PLoS One. 9:105370; Pazmandi K, et al. 2014. Free Radic Biol Med. 77:281; Fontana C, et al. 2016. J Biol Chem. 291: 7727-7741; Zhu J, et al. 2016. Sci Rep. 6:27136; Novkovic M, et al. 2016. PLoS Biol. 14: 1002515; Espinosa-Cueto P, et al. 2017. PLoS One.. 10.1371/journal.pone.0182126; Platteel A, et al. 2017. Vaccine.. 10.1016/j.vaccine.2017.10.044; Porrello A, et al. 2018. Nat Commun. 9:1988; Dhandapani R, et al. 2018. Nat Commun. 9:1640
• CD80 (clone 16-10A1), Phycoerythrin, Biolegend #104708
AB_313129
Rozanski C, et al. 2011. J Exp Med. 208:1435; Simmons D, et al. 2012. J Immunol. 188:3116; Marshall N, et al. 2012. Cancer Res. 72:581; Jeisy-Scott V, et al. 2012. J Virol. 86:10988; Wong E, et al. 2012. J Immunol. 189:5667; Nagaoka M, et al. 2014. J Immunol. 193:2812; White C, et al. 2015. J Immunol. 194:697; Sharma S, et al. 2015. J Immunol. 194:5529; Koyama M, et al. 2015. J Exp Med. 212: 1303 - 1321; Tateishi R, et al. 2015. PLoS One. 10: e0141650; Sun L, et al. 2015. Sci Rep. 5: 14871; Sebina I, et al. 2016. PLoS Pathog. 12:e1005999

• CD86 (clone GL-1), Fluorescein Isothiocyanate, Biolegend #105006
AB_313149
Benhamron S, et al. 2012. PLoS One. 7:e35602; Tsang J, et al. 2011. Int Immunopharmacol. 11:604; Liang Y, et al. 2014. J Immunol. 192:1277; DeFalco T, et al. 2014. Proc Natl Acad Sci U S A. 111:2384; Rezende R, et al. 2015. Nat Commun. 6: 8726; Nocera D, et al. 2016. J Immunol. 196: 2860 - 2869; Fontana C, et al. 2016. J Biol Chem. 291: 7727-7741; Nechama M, et al. 2018. Nat Commun. 9:1603; Habib S, et al. 2018. Infect Immun. 86:e00019; Lin SY, et al. 2017. Autophagy. 14:778; Matundan H, et al. 2019. J Virol. 93; Takeda Y, et al. 2018. Innate Immun. 24:323
• CD206 (clone C068C2), Phycoerythrin-Cyanine7, Biolegend #141720
AB_2562248
Scholz A, et al. 2016. EMBO Mol Med. 8: 39 - 57; Upadhya R, et al. 2016. MBio. 7: 00547-16; Xiang W, et al. 2018. Nat Commun. 9:2574; Nagai Y, et al. 2019. Front Immunol. 10:174; Knox T, et al. 2019. Sci Rep. 9:6136; Chhatbar C et al. 2018. Cell reports. 25 (1):118-129; Zhu Y et al. 2017. The Journal of Neuroscience. 37(9):2362-2376; Gubin MM, et al. 2018. Cell. 175:1014; Ravussin A, et al. 2018. Cell Rep. 24:1085; Heinen A, et al. 2019. Mol Ther. 27:46:00; Zhang L, et al. 2019. Front Immunol. 1.381944444; Nowak W, et al. 2019. EBioMedicine. 50:290
• iNOS/NOS2 (clone CXNFT), Alexa Fluor 488, Thermo Fisher Scientific #53-5920-82
AB_2574423
PMID:24938744; PMID:25370534; PMID:28575659; PMID:30552021
• phospho-STAT1(Y701) (clone 4a), Phycoerythrin, BD Bioscience #612564
AB_399855
PMID:28552348; PMID:30173916; PMID:30314758; PMID:32102981

WB
• α-actinin (polyclonal), Cell Signaling Technology #3134
AB_2223798
PMID:28725915
• STAT1 (polyclonal), Cell Signaling Technology #9172S
AB_2198300
PMID:24108072; PMID:29113698; PMID:29657129; PMID:30318148; PMID:30423296; PMID:30745181; PMID:30827685; PMID:30952515; PMID:31399282; PMID:31429823; PMID:31917470; PMID:31990070
• phospho-STAT1(Y701) (clone 58D6), Cell Signaling Technology #9167S
AB_561284
PMID:28575659; PMID:28810147; PMID:29045901; PMID:29113698; PMID:29129717; PMID:29544097; PMID:29562202; PMID:29657129; PMID:29691338; PMID:30173917; PMID:30193849; PMID:30282041; PMID:30423296; PMID:30454647; PMID:30472208; PMID:30503285; PMID:30552022; PMID:30566882; PMID:30645975; PMID:30776676; PMID:30930146; PMID:30952515; PMID:31091449; PMID:31429823; PMID:31597089; PMID:31619669; PMID:31665637; PMID:31708446; PMID:31715132; PMID:31722205; PMID:31917470; PMID:32057296
• horseradish peroxidase conjugated anti-rabbit IgG, Vector Laboratories #PI-1000-1
AB_2336198
PMID:24877622; PMID:28613156; PMID:28725178; PMID:28833137; PMID:28842413; PMID:29268097; PMID:29449801; PMID:29727617; PMID:29747224; PMID:29793154; PMID:29988555; PMID:30082068; PMID:30355627; PMID:30554945; PMID:30618563; PMID:30982770; PMID:31188470; PMID:31386177; PMID:31473781; PMID:31649239

Eukaryotic cell lines

Policy information about cell lines

Cell line source(s)	ATCC L-929 (ATCC CCL-1) for production of conditioned medium.
Authentication	L-929 were tested for their ability to induce macrophage differentiation in mouse bone marrow cells.
Mycoplasma contamination	Negative
Commonly misidentified lines (See ICLAC register)	L-929 cells is not a commonly misidentified line according to the current ICLAC register (Version 9).

Animals and other organisms

Policy information about studies involving animals; ARRIVE guidelines recommended for reporting animal research

Laboratory animals	C57BL/6J mice, STAT1-/- mice, IFNAR1-/- mice, IFNβ -/- mice, P2Y1-/- mice (all on C57BL/6J background) and RAGE-/- mice (on A/J background) were maintained in a specific pathogen-free environment (animals of all genotypes were males, 8-12 week old).<br><br>Germ-free and monocolonized Swiss Webster mice were age- and sex-matched (males and females of 10-16 weeks of age) and were housed in sterile flexible film isolators.<br><br>Housing conditions fo all animals: circadian light/dark (12h/12h) cycle, 22±2°C ambient temperature, 45-60% humidity, free access to food and water.

Wild animals | The study did not involve wild animals.

Field-collected samples | The study did not involve sample collected from the field.

Ethics oversight | All studies with mice were approved by the State Investigation Office of Rhineland-Palatinate, the Institutional Animal Care and Use Committee (IACUC) of Boston University and were in accordance with the guidelines of the U.S. National Institutes of Health, the German animal protection act, and the Federation of European Laboratory Animal Science Associations, directive 2010/63/EU of the European Parliament and of the Council of the European Union.

Note that full information on the approval of the study protocol must also be provided in the manuscript.

Flow Cytometry

Plots

Confirm that:

- [x] The axis labels state the marker and fluorochrome used (e.g. CD4-FITC).
- [x] The axis scales are clearly visible. Include numbers along axes only for bottom left plot of group (a 'group' is an analysis of identical markers).
- [x] All plots are contour plots with outliers or pseudocolor plots.
- [x] A numerical value for number of cells or percentage (with statistics) is provided.

Methodology

Sample preparation	Cells were washed in ice-cold sterile PBS (300g, 5 min, 4°C) and stained for 30 min with fixable viability dye eFluor 780 (Thermo Fisher Scientific) using heat-killed (1 min, 65°C) cells as positive controls. Next, cells were washed twice with FACS buffer (0.25% (w/v) BSA, 0.02% (w/v) sodium azide, 2 mM EDTA in sterile PBS), preincubated for 15 min with anti-CD16/CD32 Fc-block antibody (10 µg/ml) in FACS buffer followed by 30 min incubation with fluorescence dye-conjugated, anti-mouse antibodies or corresponding isotype controls.<br><br>For intracellular staining, the cells were fixed/permeabilized with Cytofix/Cytoperm (BD Bioscience) for 20 min followed by washing with Perm/Wash buffer (BD Bioscience) and incubation with antibodies or corresponding isotype controls for 30 min on ice. Samples were washed twice with Perm/Wash buffer and re-suspended in FACS buffer.<br><br>For phospho-flow cytometry, cells were washed with FACS buffer after stimulation, fixed for 20 min with Cytofix (BD Bioscience), washed with FACS buffer, re-suspended in pre-cooled (-20°C) Perm III buffer (BD Bioscience) and incubated overnight at -20°C. Thereafter, samples were washed again with FACS buffer, incubated for 15 min on ice with anti-CD16/CD32 blocking antibody (BioLegend) before antibodies against phospho-STAT1(Y701) and antibodies for surface markers were added for additional 30 min. Cells were washed with Perm/Wash and re-suspended in FACS buffer.
Instrument	BD Canto II
Software	BD DIVA for acquisition<br>FlowJo V10 for data analysis
Cell population abundance	At least 50,000 cells of the population of interest were acquired per sample - to determine absolute numbers counting beads were included
Gating strategy	Cells were gated for singlet events via FSC-A vs FSC-H.<br>Gate for live cells was selected based on unstained and positive (heat-killed) cells.<br>All further gates were set based on isotype and/or fluorescence minus one controls (below 1% positive cells within these controls).

- [x] Tick this box to confirm that a figure exemplifying the gating strategy is provided in the Supplementary Information.
